# Clinical identification of the stimulus intensity to measure temporal summation of second pain

**DOI:** 10.1038/s41598-022-17171-6

**Published:** 2022-07-28

**Authors:** Daisuke Moriguchi, Shoichi Ishigaki, Xiaoyu Lin, Kotaro Kuyama, Yukiko Koishi, Ryota Takaoka, Peter Svensson, Hirofumi Yatani

**Affiliations:** 1 Department of Fixed Prosthodontics, Osaka University Graduate School of Dentistry, 1-8, Yamadaoka, Suita, Osaka 565-0871 Japan; 2 Department of Dentistry and Oral Health, Section for Orofacial Pain and Jaw Function, Aarhus University, Aarhus, Denmark; 3 Faculty of Odontology, Malmo University, Malmo, Sweden

**Keywords:** Neuroscience, Physiology, Diseases, Medical research, Neurology, Risk factors, Signs and symptoms

## Abstract

Temporal summation of second pain (TSSP) has been suggested as a psychophysical index for central sensitization, one of the critical mechanisms in the chronification of pain. However, there is no gold standard for protocols to measure TSSP. The purpose was to establish the stimulus intensity for measuring TSSP. Female patients with chronic myofascial temporomandibular disorders pain (n = 16) and healthy female volunteers with no pain (n = 15) participated. Pain thresholds (PT °C) were measured, and repetitive heat stimuli at three stimulus intensities (PT °C, PT + 1 °C, PT + 2 °C) were applied. TSSP parameters were quantified as TSSP magnitude (TSm) and TSSP frequency (TSf). In healthy female volunteers, pain ratings significantly decreased at PT °C (p < 0.050), besides TSm and TSf at PT + 2 °C were significantly higher than those at PT °C (p < 0.025). In chronic pain patients, pain ratings significantly increased at PT + 1 °C and PT + 2 °C (p < 0.050). At PT + 2 °C, TSm and TSf in chronic pain patients were significantly higher than those in healthy volunteers (p < 0.050). It could be helpful to measure TSSP with the stimulus intensity adjusted individually to the patient’s pain thresholds + 2 °C for assessing central sensitization.

## Introduction

Central sensitization (CS) is defined as “Increased responsiveness of nociceptive neurons in the central nervous system to their normal or subthreshold afferent input”^1^. CS has been proposed as one of the critical mechanisms in many chronic pain-related conditions that have a disparity between the magnitude of tissue damage and self-reported pain intensity, which cannot be readily explained^2,3^. CS seems to be essential to understand in the chronification of pain^4^. Yunus et al. have first proposed “central sensitivity syndrome” to characterize these CS-related disorders^5,6^. Recently, the National Institutions of Health has proposed the term “chronic overlapping pain conditions” to recognize the concept of coexisting pain conditions as a set of disorders that may share a common mechanism and includes^7,8^, fibromyalgia, irritable bowel syndrome, vulvodynia, myalgic encephalomyelitis/chronic fatigue syndrome, interstitial cystitis/painful bladder syndrome, endometriosis, chronic tension-type headache, migraine headache, and chronic lower back pain^2,9^, not limited to temporomandibular disorders. Thus it is imperative to establish a way to detect CS^2,3,10,11^. To diagnose and treat chronic pain conditions, we must examine whether this root cause is CS. If CS causes chronic pain conditions, we need to approach the central nervous system^10^.

Dynamic Quantitative Sensory Test (dynamic QST) could be a valuable technique to assess CS^9,11–19^. It is a way to evaluate the excitability of different pain pathways/mechanisms using various stimulus modalities and quantify pain objectively^12–15,20^. Dynamic QST can measure a Temporal Summation of Second Pain (TSSP) phenomenon, which has long been proposed as an essential modality for functional evaluation of CS^2,10,11^.

Existing protocols for measuring TSSP usually apply a fixed stimulus intensity in an arbitrary way^12,13^. More recently, individualized protocols suited for within-individual monitoring have been developed^14,16,21,22^. The stimulus intensity is determined to assure a mild painful sensation at the start of the stimulus for measuring TSSP. It might be logical to adjust the stimulus intensity individually because the patients’ pain thresholds differ from individual to individual^23–26^. Although there was no evidence to set this stimulus intensity, an earlier study applied individual’s pain thresholds + 2 °C as the stimulus intensity for the individualized protocol^22^, another study applied 1.25 times individual’s pain thresholds as the stimulus intensity^27^, and another study applied a mild painful sensation adjusted individually^14,16,21^. However, there is no gold standard for protocols to set the stimulus intensity for measuring TSSP.

This study’s objective was to establish the stimulus intensity for the best measuring TSSP. The null hypothesis was as follows: 1. In healthy volunteers with no pain, TSSP with the stimulus intensity adjusted at individual’s pain thresholds + 2 °C would not be measured more significantly than that with the stimulus intensity adjusted at individual’s pain thresholds and the stimulus intensity adjusted at individual’s pain thresholds + 1 °C; and 2. TSSP differences between chronic pain patients and healthy volunteers with no pain would not be observed with the stimulus intensity adjusted at individual’s pain thresholds + 2 °C more significantly than with the stimulus intensity adjusted at individual’s pain thresholds and the stimulus intensity adjusted at individual’s pain thresholds + 1 °C. To examine null hypothesis 1, TSSP was compared across the stimulus intensities in healthy volunteers with no pain. To examine null hypothesis 2, a case–control study of healthy volunteers with no pain and temporomandibular disorders patients with chronic pain was conducted.

## Methods

### Experimental design

**Figure 1:**
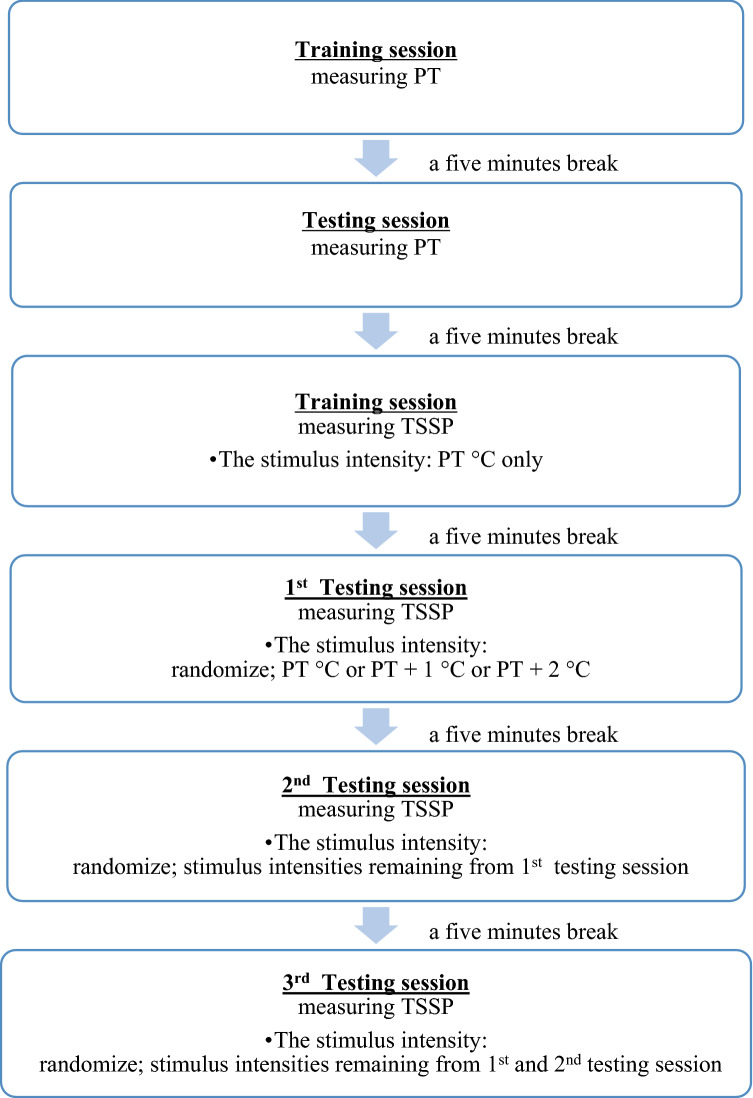
Flow chart of a protocol for measuring PT and TSSP.

The pain thresholds of each participant (PT °C) was determined, and then TSSP was measured using three stimulus intensities (PT °C, PT + 1 °C, PT + 2 °C) (Fig. 1). Heat stimuli were applied to the non-dominant thenar eminence at the orientation and training session, and heat stimuli were applied to the dominant thenar eminence at the testing session.

Participants were sitting relaxed on a chair in a quiet room at from 20 to 24 °C. The test was performed using a computerized thermal sensory stimulator (PATHWAY; Medoc, Ramat Yishai, Israel) via a thermode (Advanced Thermal Stimulator model 30 × 30 mm^2^; Medoc, Ramat Yishai, Israel). A computer controled the stimulus delivered with PATHWAY. The temperature span is from − 10 to 55 °C. When the TSSP was measured, the experimental pain was rated continuously using a computerized visual analogue scale (CoVAS; Medoc, Ramat Yishai, Israel). It is an electronic visual analog scale (VAS) used to rate the pain intensity for each thermal pulse. Participants were asked to rate the perceived pain during stimulus application by moving the CoVAS slider continuously. The extreme left on the 100 mm CoVAS was labeled “no pain” and the extreme right was “most pain imaginable”.

### Participants

The sample size was calculated by setting risks for type 1 and type 2 errors of 5% and 20%, respectively. Mean and standard deviation of experienced pain were obtained from a pilot study. This pilot study included 11 healthy volunteers with no pain and 8 patients with chronic pain. In healthy volunteers, a mean of TSSP magnitude and TSSP frequency was 1.4 ± 3.2 and 1.4 ± 1.6 respectively. In patients with chronic pain, a mean of TSSP magnitude and TSSP frequency was 26.0 ± 18.0 and 4.0 ± 0.8, respectively.

**Figure 2:**
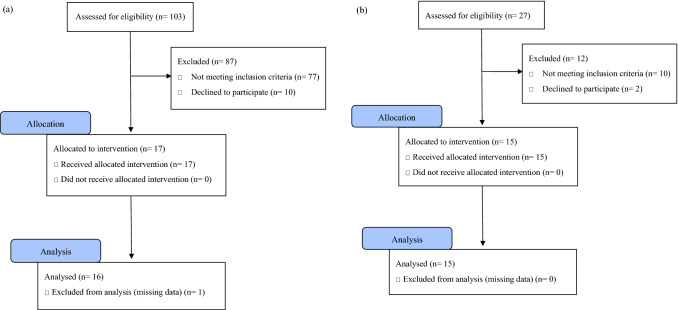
Flow diagram of participants. (**a**) Chronic pain patients, (**b**) healthy volunteers with no pain.

Periods of recruitment and data collection were from April 2018 to August 2019. As a patient group, 103 consecutive participants were recruited from outpatients at the Department of Fixed Prosthodontics at Osaka University Dental Hospital. As a control group, 27 participants were recruited from Osaka University Dental Hospital staff. The criteria were shown in Table 1. Out of 103 participants recruited as a patient group, 16 female chronic pain patients were identified. Out of 27 participants recruited as a control group, 15 female healthy volunteers with no pain were identified. Flow diagrams of participants can be found in Fig. 2. They were diagnosed following the ICOP^28^ and the DC/TMD^29^ by two instructors from the Japanese Society for Temporomandibular Joint and the Japanese Society of Orofacial Pain. A Global Severity Index is one of the global indices of distress associated with the Symptom Checklist-90-Revised (SCL-90-R)^30,31^ and the single indicator of the current level or depth of the disorder. In the SCL-90-R, raw scores are converted to normalized T-scores using the norm group appropriate for the person being examined. T-scores are characterized by a distribution with a mean of 50 and a standard deviation of 10. The history of pain was obtained using the Patient Health Questionnaire-15 (PHQ-15)^32^ and the TMD Pain Screener^33^.

**Table 1:** The criteria of two groups; chronic pain patients and healthy volunteers with no pain.

	Inclusion criteria
Chronic pain patients	Those diagnosed with any of the following (1) Chronic frequent primary myofascial orofacial pain (2) Chronic highly frequent primary myofascial orofacial pain (3) Chronic frequent primary temporomandibular joint pain (4) Chronic highly frequent primary temporomandibular joint pain
Healthy volunteers with no pain	(1) Those with good health status with no self-reported diseases (2) Those with a Global Severity Index < 60 (3) Those who had no pain for four weeks before this test (including back pain, stomach pain, pain in arms or legs, menstrual cramps, headache, chest pain, masticatory muscle pain, and temporomandibular joint pain)

The general inclusion criteria of participants were: (1) willing to participate and give informed consent; (2) above 20 years of age. The general exclusion criteria were: (1) individuals unable to communicate or follow instructions; (2) use of narcotics, antidepressants, and psychotropics; and (3) individuals with paralysis or a skin disorder at hand.

### Orientation and training session for measurements of pain thresholds

A standard script was read for each participant before measuring the pain thresholds. Heat stimuli were applied to the non-dominant thenar eminence. The starting temperature was 32 °C, and then the temperature of the stimuli was changed at several ascending and descending sets of stimuli. Particular care was taken to instruct the participants and ensure they understood the measurement procedure well. All participants received training until they were familiar with the test protocol.

### Testing session for measurements of pain thresholds

Heat stimuli were applied to the dominant thenar eminence. Pain thresholds (PT °C) were measured following the instructions developed by the German Research Network on Neuropathic Pain (DFNS). All pain thresholds were obtained with ramped stimuli (1 °C/s) terminated when the participant pressed a button. During the experiment, the participants could not watch the computer screen.

### Orientation and training session for measurements of TSSP

Again, a standard script was read to each participant, which defined the VAS scale and instructed each participant to rate the slow, burning sensation from each pulse (second pain), not the prickly sensation immediately felt at the delivery of each pulse (first pain). We alerted participants to expect a delay between the delivery of each pulse and the perception of the second pain.

Heat stimuli were applied to the non-dominant thenar eminence, while participants rated the experimental pain using the CoVAS with the other hand. Ten consecutive heat stimuli at PT °C with an interstimulus interval of two seconds were applied. As for the instructions, particular care was taken to ensure that participants understood the measurement procedure well, and they had training repeatedly until the TSSP protocol was familiar to them.

### Testing session for measurements of TSSP

Heat stimuli were applied to the dominant thenar eminence, while participants rated the experimental pain using the CoVAS with the other hand. Ten consecutive heat stimuli with an interstimulus interval of 2 s at three stimulus intensities (PT °C, PT + 1 °C, PT + 2 °C) were applied. The stimulus intensity applied was determined randomly. Neither participants nor experimenters knew which stimulus intensity was applied.

Participants had a 5 minutes break between each trial to avoid any after-sensations or ongoing pain before the subsequent trial. The measurement was blinded for examiners.

### Calculations of TSSP parameters

Pain Intensity (PI_n_) was calculated by integrating the values of pain ratings from the nth stimulus to the n + 1th stimulus. TSSP parameters (TSSP magnitude, TSSP frequency) were calculated using the PI and defined. TSSP magnitude was an index for evaluating the magnitude we observed a temporal summation phenomenon. TSSP frequency was an index for evaluating the number of times we observed a temporal summation phenomenon.

$${\text{TSSP magnitude }}\left( {{\text{TSm}}} \right):{\text{ PI}}_{{{\text{max}}}} - {\text{ PI}}_{{1}} ({\text{VAS}} \times {\text{second}}),$$

$${\text{TSSP frequency }}\left( {{\text{TSf}}} \right):{\text{ Numbers of times P}}_{{{\text{n }} + { 1}}} > {\text{ PI}}_{{\text{n}}} \left( {{\text{times}}} \right).$$

### Assessments of biopsychosocial functions

Jaw pain was assessed with a visual analog scale. Somatic symptoms were assessed with the PHQ-15^32^. The illness period (month) was assessed as the pain period before this test.

Past pain experiences and negative thoughts or feelings about pain were assessed with the Pain Catastrophizing Scale (PCS)^34^. The self-efficacy belief in individuals with pain was assessed with the pain self-efficacy questionnaire (PSEQ)^35^. Somatization (SOM), obsessive–compulsive (O–C), Anxiety (ANX), and depression (DEP) were assessed with the T-scores of the SCL-90-R^30,31^.

### Data reductions and statistical methods

#### TSSP response patterns at each stimulus intensity in healthy volunteers with no pain and chronic pain patients

All statistical data were entered and analyzed using a dedicated statistical software (SPSS, Version 21.0; IBM, Armonk, NY, USA). Generalized Linear Mixed Models were conducted to examine the effect of repetitive stimuli on PI. The number of stimuli and age were used as covariates. Log (PI + 1) was used as the dependent variable. The variable conversion was performed using log because PI did not have a normal distribution. The significance level was set at α = 0.050.

#### Differences in TSSP parameters across the stimulus intensities in healthy volunteers with no pain

Wilcoxon signed-rank test was conducted to examine the differences in TSm and TSf between PT °C and PT + 1 °C, PT + 1 °C and PT + 2 °C. The significance level corrected with Bonferroni procedures was set at α = 0.025.

#### Differences in TSSP parameters between chronic pain patients and healthy volunteers with no pain

Mann–Whitney’s U test was conducted to examine the differences in TSm and TSf between groups. The significance level was set at α = 0.050.

### Ethics approval and consent to participate

The present research complied with the STROBE statement. This study was conducted in accordance with the guidelines of the Declaration of Helsinki and the principles of good practice and performed with the approval of the Osaka University Graduate School of Dentistry and the Ethics Review Committee of the Dental Hospital (H29-E43). Informed consent for participating in this study and publication was obtained from every participant.

## Results

### Descriptive data

This study included 15 healthy volunteers with no pain and 16 chronic pain patients (Raw data of TSSP measurement can be found in supplementary information). Overall, significant group differences were found for age, pain thresholds, jaw pain, PHQ-15, illness period, SOM, O-C, DEP, ANX, and PCS (all p < 0.050, the details of p-value were as Table 2).

**Table 2:** Descriptive data of participants.

	Healthy volunteers with no pain n = 15	Chronic pain patients n = 16	p-value
Age	27 ± 2.6	51 ± 13	< 0.001
PT °C	47 ± 1.0	48 ± 1.2	0.045
PT + 1 °C	48 ± 1.0	49 ± 1.2	0.045
PT + 2 °C	49 ± 1.0	50 ± 1.2	0.045
Jaw pain (0–100)	0.00 ± 0.00	51 ± 19	< 0.001
PHQ-15 (0–30)	0.20 ± 0.56	7.8 ± 5.4	< 0.001
Illness period (month)	0.00 ± 0.00	21 ± 53	< 0.001
SOM (0–100)	40 ± 7.5	60 ± 8.3	< 0.001
O-C (0–100)	48 ± 9.4	59 ± 7.8	0.002
DEP (0–100)	43 ± 8.5	59 ± 8.0	< 0.001
ANX (0–100)	39 ± 4.9	51 ± 12	0.001
PCS (0–52)	6.4 ± 8.6	24 ± 12	< 0.001
PSEQ (0–60)	42 ± 9.6	31 ± 17	0.049

Data are reported as mean ± standard deviation (S.D.).

*PHQ-15* patient health questionnaire-15, *SOM* somatization, *O–C* obsessive–compulsive, *DEP* depression, *ANX* anxiety, *PCS* pain catastrophizing scale, *PSEQ* pain self-efficacy questionnaire.

### TSSP response patterns in healthy volunteers with no pain

**Figure 3:**
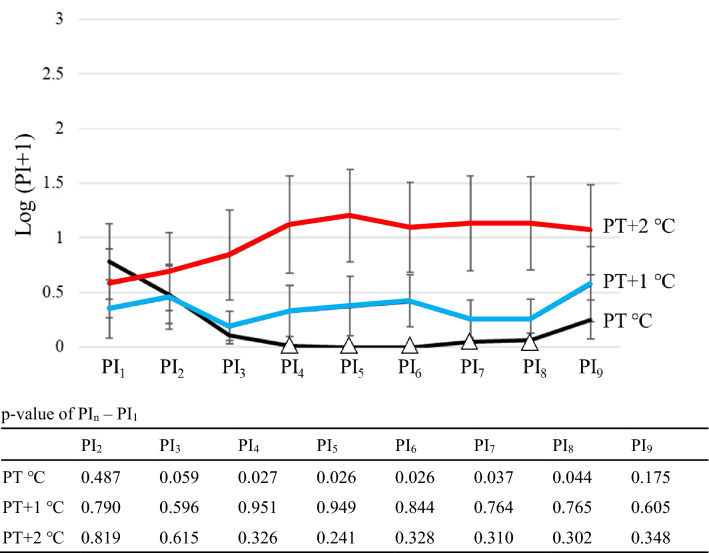
TSSP response patterns in healthy volunteers with no pain (female only: n = 15). Open triangle showed that PI_n_ was significantly lower than PI_1_. Pain Intensity (PI_n_) was calculated by integrating the values of pain ratings from the nth stimulus to the n + 1th stimulus.

At PT °C, PI_4-8_ was significantly lower than PI_1_ (p-value; 0.487 at PI_2_, 0.059 at PI_3_, 0.027 at PI_4_, 0.026 at PI_5_, 0.026 at PI_6_, 0.037 at PI_7_, 0.044 at PI_8_, 0.175 at PI_9_) (Fig. 3). At PT + 1 °C, PI_2–9_ did not change significantly compared to PI_1_ (p-value; 0.790 at PI_2_, 0.596 at PI_3_, 0.951 at PI_4_, 0.949 at PI_5_, 0.844 at PI_6_, 0.764 at PI_7_, 0.765 at PI_8_, 0.605 at PI_9_) (Fig. 3). At PT + 2 °C, PI_2–9_ did not change significantly compared to PI_1_ (p-value; 0.819 at PI_2_, 0.615 at PI_3_, 0.326 at PI_4_, 0.241 at PI_5_, 0.328 at PI_6_, 0.310 at PI_7_, 0.302 at PI_8_, 0.348 at PI_9_) (Fig. 3).

### Differences in TSSP parameters across the stimulus intensities in healthy volunteers with no pain

TSm and TSf at PT + 2 °C were significantly higher than those at PT °C (p-value was 0.021 and 0.011, respectively) (Table 3). There were no significant differences between PT °C and PT + 1 °C in TSm and TSf (p-value was 0.075 and 0.066, respectively).

**Table 3:** Outcome data of TSSP parameters in Healthy volunteers with no pain and Chronic pain patients.

	Healthy volunteers with no pain n = 15			Chronic pain patients n = 16		
	PT °C	PT + 1 °C	PT + 2 °C	PT °C	PT + 1 °C	PT + 2 °C
TSm	0.23 ± 0.74	4.6 ± 9.2	14 ± 24	15 ± 37	19 ± 33	49 ± 63
TSf	0.40 ± 1.1	1.1 ± 1.9	1.9 ± 2.1	1.2 ± 1.5	2.0 ± 2.6	3.9 ± 2.3

Mean ± S.D.

TSm: PI_max_ − PI_1_ (VAS × second).

TSf: Numbers of times P_n + 1_ > PI_n_ (times).

### TSSP response patterns in chronic pain patients

**Figure 4:**
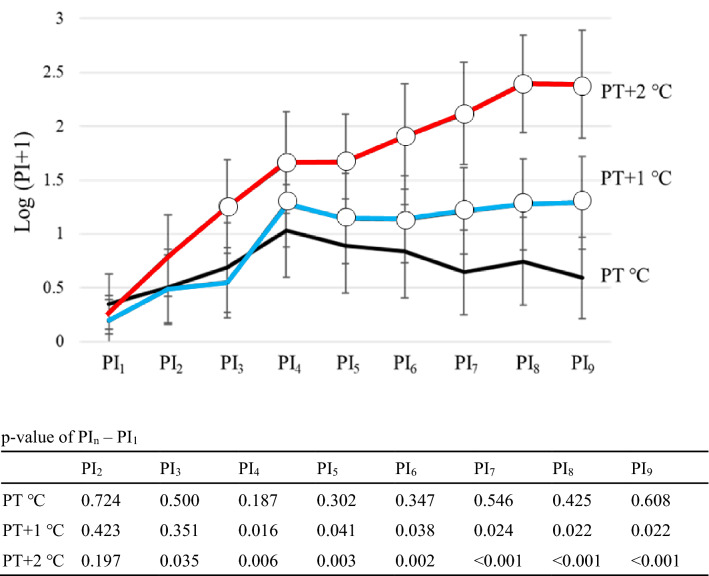
TSSP response patterns in chronic pain patients (female only; n = 16). Open circle showed that PI_n_ was significantly higher than PI_1_. Pain Intensity (PI_n_) was calculated by integrating the values of pain ratings from the nth stimulus to the n + 1th stimulus.

At PT °C, PI_2-9_ did not change significantly compared to PI_1_ (p-value; 0.724 at PI_2_, 0.500 at PI_3_, 0.187 at PI_4_, 0.302 at PI_5_, 0.347 at PI_6_, 0.546 at PI_7_, 0.425 at PI_8_, 0.608 at PI_9_) (Fig. 4). At PT + 1 °C, PI_4-9_ was significantly higher than PI_1_ (p-value; 0.423 at PI_2_, 0.351 at PI_3_, 0.016 at PI_4_, 0.041 at PI_5_, 0.038 at PI_6_, 0.024 at PI_7_, 0.022 at PI_8_, 0.022 at PI_9_) (Fig. 4). At PT + 2 °C, PI_3-9_ was significantly higher than PI_1_ (p-value; 0.197 at PI_2_, 0.035 at PI_3_, 0.006 at PI_4_, 0.003 at PI_5_, 0.002 at PI_6_, < 0.001 at PI_7_, < 0.001 at PI_8_, < 0.001 at PI_9_) (Fig. 4).

### Differences in TSSP parameters between chronic pain patients and healthy volunteers with no pain

There were significantly higher TSm and TSf in chronic pain patients at PT + 2 °C (p-value was 0.041 and 0.027, respectively) (Table 3). There were no significant differences by group in TSm at PT °C and PT + 1 °C (p-value was 0.119 and 0.318, respectively). Also, there were no significant differences by group in TSf at PT °C and PT + 1 °C (p-value was 0.202 and 0.423, respectively).

## Discussion

To assess CS, a gold standard for TSSP protocols is required. This is the first study that has examined the clinical identification of TSSP protocols with the stimulus intensity adjusted individually to the patient’s pain thresholds. This study has provided an evidence for a practical clinical paradigm to set the stimulus intensity for measuring TSSP.

In this study, the null hypothesis was rejected. Therefore: 1. In healthy volunteers with no pain, TSSP was measured at PT + 2 °C more significantly than at PT °C and PT + 1 °C; and 2. TSSP differences between chronic pain patients and healthy volunteers with no pain were observed at PT + 2 °C more significantly than at PT °C and PT + 1 °C.

Wind-up, TSSP, and CS share common characteristics and their definitions are complex, but they should be distinguished. TSSP refers to increased pain perception from repetitive, noxious stimuli^4,10,11^. The neurophysiological underpinnings of this phenomenon probably result from enhancements of a central *N*-methyl-d-aspartate (NMDA) receptor mechanism within the spinal dorsal horn (brain stem) nociceptive neurons, which is termed “wind-up”^4^. The wind-up process measured from dorsal horn wide-dynamic range neurons in animals is a progressive increase in neuronal output during the course of a train of identical afferent nociceptive stimuli^11^. This repeated high intensity afferent barrage will cause the increased neuronal output to last after the end of the repeated stimuli^11^. In humans, psychological or electrophysiological responses are used as proxies for the initial part of the wind-up process^11^. This phase translates into TSSP, which is so-called wind-up like pain^11^. TSSP is an essential index for evaluating CS.

Wind-up has been first proposed as the increased firings of excitatory postsynaptic potential (EPSP) within the dorsal horn (brain stem) induced by repetitive electrical stimuli of peripheral C-fibers at train frequencies of more than 0.33 Hz^36^. The pathway of input of nociceptive impulses in the trigeminal nervous system is different from that in the spinal nervous system^37^. Nociceptive impulses arising from the face and oral structures are carried by the primary afferent neuron of the mandibular or maxillary division of the trigeminal nerve (V2, V3) through the gasserian ganglion into the subnucleus caudalis region of the trigeminal spinal tract. It synapses with a second-order neuron, and the input is carried to the thalamus and the cortex^37^. However, there has been no evidence that the skin overlying the masseter of TMD pain patients presents peripheral sensitization. Future studies should be assessed through a stimulus applied to the muscle and not the surface.

TSSP magnitude (PI_max_ − PI_1_) is popular and useful for a TSSP parameter, but this could not reveal a process of TSSP. There is a risk that TSSP magnitude could be the same despite the different TSSP processes (whether a subjective pain intensity is gradually increased by repeated stimulation or a subjective pain intensity is momentarily increased by a single stimulation). So, in this study, TSSP frequency (Numbers of times P_n + 1_ > PI_n_) was added for TSSP parameters.

The stimulus intensity was needed to ensure a painful sensation at the start of the stimulus for measuring TSSP, so the stimulus intensity in this study was set to PT °C or higher. To investigate at what higher stimulus intensities TSSP should cease to occur due to a ceiling effect, it is needed to measure TSSP at PT + 3 °C or higher. However, in the pilot study, five participants were tested at PT + 3 °C, and they felt too much pain that the test was discontinued. Therefore, measuring TSSP at PT + 3 °C might be unethical. This study’s results of TSSP in healthy volunteers with no pain revealed that PT + 2 °C was enough for the stimulus intensity for measuring TSSP, as described later. Thus, this study did not measure TSSP at PT + 3 °C or higher.

When an interstimulus interval is more than 0.33 Hz, a wind-up phenomenon could occur, that is, TSSP could occur^36^. So, an interstimulus interval should be more than 0.33 Hz for measuring TSSP. In this study, an interstimulus interval was set to two seconds because most of the earlier studies set an interstimulus interval to 2 s^14,16,21,22^.

There is no gold standard for protocols to set the number of stimuli for measuring TSSP. Earlier studies set the number of stimuli to 10^14,16^ or 15^21^ or 20^22^ etc. in an arbitrary way. In an earlier study in which the number of stimuli was set to 20, TSSP was clearly measured at 10 stimulus times^22^. Of course, TSSP was clearly measured in earlier studies when the number of stimuli was set to 10^14,16^. In this study, the number of stimuli was set to 10 because TSSP was measured clearly when 10 consecutive heat stimuli were applied in the pilot study. Future studies are needed to identify the appropriate numbers of stimuli for measuring TSSP.

In this study, nociceptive stimuli were applied to the spinal nervous system (hands) to exclude potential confounding from peripheral sensitization and to assess CS more directly. In many studies, such as the Orofacial Pain: Prospective Evaluation and Risk Assessment (OPPERA)^38^, nociceptive stimuli were also applied to the spinally innervated areas in TMD patients.

TSSP could be facilitated using various noxious stimuli, such as electrical, mechanical, and heat stimuli^11,17^. There are equally valid and reliable techniques to measure TSSP in orofacial pain conditions^39^, and it is not clear what is the advantage of the proposed one, in particular, because there is no comparator protocol. TSSP depends on spinal processes driven by the activation of C-fibers. Participants must rate the second pain evoked by C-fibers activation and not the first pain evoked by A-delta-fibers activation^40^.

### TSSP in healthy volunteers with no pain

Generalized Linear Mixed Models to examine TSSP response patterns at each stimulus intensity revealed that TSSP decreased significantly at PT °C. This result suggests that measuring TSSP at PT °C could not avoid a floor effect. PT °C was too weak for the stimulus intensity to measure TSSP. So, the stimulus intensity should be set to more than PT °C to assure a mild painful sensation that could avoid a floor effect. Although there has been no clear rationale, earlier studies set the stimulus intensity to more than PT °C. Zhou et al. set the stimulus intensity to PT + 2 °C^22^. Janal et al. set the stimulus intensity to a magnitude of TSSP adjusted to VAS 0/100 or more^21^. Kong and Mackey et al. set the stimulus intensity to a magnitude of TSSP adjusted to VAS 30/100 to 70/100^14,16^.

TSm and TSf at PT + 2 °C increased significantly more than those at PT + 1 °C. This result suggests that the stimulus intensity should be set to PT + 2 °C rather than PT + 1 °C to measure TSSP more clearly.

### TSSP in chronic pain patients

Generalized Linear Mixed Models to examine TSSP response patterns at each stimulus intensity revealed that TSSP did not increase significantly at PT °C and that TSSP increased significantly at PT + 1 °C and PT + 2 °C. TSSP response patterns in chronic pain patients were higher than those in healthy volunteers with no pain at all stimulus intensities. Besides, at PT + 2 °C, TSm and TSf were significantly higher in chronic pain patients than in healthy volunteers with no pain. These results might indicate that the differences between chronic pain patients and healthy volunteers with no pain could be observed only at PT + 2 °C. The stimulus intensity should be individually adjusted to 2 °C above the patient’s pain thresholds to measure TSSP. Earlier studies do not contrast with our study. Maixner et al. reported that TSSP response patterns in TMD patients with chronic pain enhanced more than those in healthy volunteers with no pain and TSm in TMD patients with chronic pain was significantly higher than that in healthy volunteers with no pain^27^. In the OPPERA study, which was a large-scale study that demonstrated pain sensitivity risk factors for chronic TMD, TSm in TMD patients with chronic pain was not significantly higher than that in healthy volunteers with no pain, but TSSP response patterns in TMD patients with chronic pain enhanced more than those in healthy volunteers with no pain^38^. But, these studies applied a fixed stimulus intensity for measuring TSSP. In studies with individualized protocols suited for within-individual monitoring, TSSP response patterns in TMD patients with chronic pain enhanced more than those in healthy volunteers with no pain at every stimulus (PT + 2 °C; first, fifth, 10th, 15th, 20th) and TSm in TMD patients with chronic pain was significantly higher than that in healthy volunteers with no pain^22^. In a case–control study with fibromyalgia patients, Staud et al. reported that TSSP response patterns in fibromyalgia patients with chronic pain was enhanced more than those in healthy volunteers with no pain and TSm in fibromyalgia patients with chronic pain was significantly higher than that in healthy volunteers with no pain^19,41,42^. In various chronic pain conditions, such as neuropathic, musculoskeletal, joint, and visceral pain, TSSP is significantly facilitated^11^. TSSP might be an index suggestive of CS since TSSP was facilitated more within CS-related disorders^11^. There is an evidence that chronic pain patients enhanced experimental pain sensitivity that appears to be facilitated by alteration in central nervous system processes that regulate the temporal processing of pain^11^.

Notwithstanding the significant advantages of the present study, a few limitations also should be acknowledged and discussed. The main limitation could be significant differences in age between healthy volunteers with no pain and chronic pain patients. Experimental data on age-related changes in TSSP are contradictory due to the methodologic differences between studies. Some studies showed that TSSP in the middle-aged and older-aged groups was significantly higher compared to that in the younger-aged group^40,43^, while some studies showed no effects of age on TSSP^44^. However, age-matched controls should be identified in future studies. Experimental pain perception was found between male and female^38^. But, in this study, participants were composed of only female. So, additional studies should investigate TSSP differences by sex.

## Conclusions

It could be helpful to measure TSSP with the stimulus intensity adjusted individually to the patient’s pain thresholds + 2 °C for assessing CS.

## Supplementary Information

Supplementary Information 1.

Supplementary Information 2.

## Data Availability

Upon a reasonable request, further information on the data set and materials is available from the corresponding author.
