# Author Correction: Clinical identification of the stimulus intensity to measure temporal summation of second pain

**DOI:** 10.1038/s41598-022-19043-5

**Published:** 2022-08-29

**Authors:** Daisuke Moriguchi, Shoichi Ishigaki, Xiaoyu Lin, Kotaro Kuyama, Yukiko Koishi, Ryota Takaoka, Peter Svensson, Hirofumi Yatani

**Affiliations:** 1 Department of Fixed Prosthodontics, Osaka University Graduate School of Dentistry, 1-8, Yamadaoka, Suita, Osaka 565-0871 Japan; 2 Department of Dentistry and Oral Health, Section for Orofacial Pain and Jaw Function, Aarhus University, Aarhus, Denmark; 3 Faculty of Odontology, Malmo University, Malmo, Sweden

Correction to: *Scientific Reports* 10.1038/s41598-022-17171-6, published online 28 July 2022

The original version of this Article contained errors in the spelling of the authors Daisuke Moriguchi, Shoichi Ishigaki, Xiaoyu Lin, Kotaro Kuyama, Yukiko Koishi, Ryota Takaoka, Peter Svensson and Hirofumi Yatani, which were incorrectly given as Moriguchi Daisuke, Ishigaki Shoichi, Lin Xiaoyu, Kuyama Kotaro, Koishi Yukiko, Takaoka Ryota, Svensson Peter and Yatani Hirofumi.

Furthermore, an incorrect email address for corresponding author Daisuke Moriguchi was quoted. Correspondence and requests for materials should be addressed to moriguchi.daisuke.dent@osaka-u.ac.jp.

The original Article has been corrected.

